# Evaluation of plasma cell sorting methods in multiple myeloma patients: flow cytometry versus magnetic beads

**DOI:** 10.1186/s12935-025-03647-8

**Published:** 2025-01-17

**Authors:** Yu Jeong Choi, Jaeguk Choi, Yehyun Kang, Saeam Shin, Seung-Tae Lee, Jong Rak Choi

**Affiliations:** 1 Department of Laboratory Medicine, Yonsei University College of Medicine, 50-1 Yonsei-ro, Seodaemun-gu, Seoul, 03722 Korea; 2 Department of Laboratory Medicine, Severance Hospital, Seoul, Korea; 3 Graduate School of Medical Science, Brain Korea 21 PLUS Project, Yonsei University College of Medicine, Seoul, Korea; 4 Dxome Co. Ltd, Seongnam-si, Gyeonggi-do Korea

**Keywords:** Plasma cell neoplasm, Plasma cell myeloma, Multiple myeloma, Cell sorting, Fluorescence-activated cell sorting, FACS, Magnetic-activated cell sorting, MACS

## Abstract

**Background:**

The prognosis of a plasma cell neoplasm (PCN) varies depending on the presence of genetic abnormalities. However, detecting sensitive genetic mutations poses challenges due to the heterogeneous nature of the cell population in bone marrow aspiration. The established gold standard for cell sorting is fluorescence-activated cell sorting (FACS), which is associated with lengthy processing times, substantial cell quantities, and expensive equipment. Magnetic-activated cell sorting (MACS) can be performed without the need for FACS equipment and allows for rapid sorting of many cells, making it a practical alternative. Our objective is to conduct a comparative analysis of these two sorting techniques to assess whether MACS can viably replace FACS in clinical applications.

**Methods:**

Plasma cell purity, fluorescence in situ hybridization (FISH), and next-generation sequencing analyses were performed on FACS- and MACS-sorted bone marrow samples from 31 PCN patients.

**Results:**

The MACS-sorted samples yielded a higher percentage of plasma cells than FACS-sorted samples under microscopy (*p* = 0.0156) and flow cytometry (*p* = 0.0313). FISH performed by two methods in 10 samples showed the same results, and the proportion of abnormal cells was significantly higher in MACS than in FACS (*p* = 0.001). Wilcoxon matched-pairs signed rank test analysis showed that the median of differences of variant allele frequency (VAF) of two methods (VAF of MACS minus VAF of FACS) in the *DNMT3A*, *TET2*, and *ASXL1* (DTA) group was − 0.006555 (*p* = 0.0020), while that in the non-DTA group was 0.002805 (*p* = 0.0019). Ten copy number variants (CNVs) were found in both FACS- and MACS-sorted samples, eight were identified only in MACS-sorted samples, and one was detected only in FACS-sorted samples.

**Conclusion:**

Our study demonstrates that MACS is a viable alternative for plasma cell sorting in bone marrow samples of patients with PCN.

**Supplementary Information:**

The online version contains supplementary material available at 10.1186/s12935-025-03647-8.

## Introduction

Multiple myeloma (MM) accounts for 1–2% of all cancers and 10% of all blood cancers [1]. Annually, approximately 160,000 people are newly diagnosed with MM, and 106,000 die worldwide from the disease [2]. Given its higher prevalence among the elderly, these numbers are anticipated to rise in the coming years. The spectrum of plasma cell neoplasms (PCNs) spans from monoclonal gammopathy of undetermined significance (MGUS), which is characterized by a bone marrow plasma cell percentage less than 10%, to MM, with values exceeding 10%. MGUS is detected in 5% of individuals aged 50 and above, with approximately 1% transitioning to MM or a related malignancy each year [3]. The risk of progression escalates with the presence of genetic abnormalities such as t(4;14), del(17p), and gain(1q) in MGUS [4]. Even after diagnosis of MM, assessment of cytogenetic risk is essential for predicting survival and establishing an effective treatment plan. Hence, identifying these mutations is crucial for accurate prognostication and timely intervention in PCN patients. However, detecting sensitive genetic mutations poses challenges due to the heterogeneous nature of the cell population in bone marrow aspiration, including a low proportion of plasma cells and specimen dilution. Consequently, the purification of plasma cells from bone marrow aspiration for genetic testing is crucial for enhancing the analytical sensitivity [5, 6].

The established gold standard for cell sorting is fluorescence-activated cell sorting (FACS), a method capable of classifying many cells into multiple populations. However, FACS is associated with lengthy processing times, substantial cell quantities, and expensive equipment. In contrast, magnetic-activated cell sorting (MACS) does not require FACS equipment and enables the efficient separation of a large number of cells, offering a practical alternative by directly labeling and sorting cells within a magnetic field. Furthermore, a recent investigation utilizing adipose tissue has demonstrated that MACS yields a superior total cell count compared to FACS [7]. We aim to conduct a comparative analysis of these two sorting techniques to assess whether MACS can viably replace FACS in clinical applications.

## Materials and methods

### Patients and samples

The study included 31 patients undergoing bone marrow evaluation for suspected PCN. Bone marrow aspirate collected in sodium heparin tubes was centrifuged at 1,000 g for 5 min to separate the cellular components. The cell pellet was separated using a serum separating tube, then aliquoted into a 15 mL tube and resuspended by vortexing before being divided in half for FACS- and MACS-sorting. Research involving human specimens complied with all relevant national regulations, institutional policies, and the tenets of the Helsinki Declaration (as revised in 2013). This study was exempted from institutional review board (IRB) review by the IRB of Severance Hospital, Seoul, Republic of Korea (4-2024-0690) because this study evaluated residual specimens.

### Plasma cell sorting using FACS

The resulting cell pellet was treated with a lysis solution by adding 10 ml to the tube, incubating in the dark for 20 min, and centrifuging at 1,000 g for 5 min. The supernatant was removed using a serum separating tube, then 11 mL of phosphate-buffered saline (PBS) was added, and the tube was centrifuged again at 1,000 g for 5 min, with the supernatant removed. This process, based on an internal protocol, was repeated twice, and finally, 1 mL of PBS was added to complete the preparation. The cells were then purified using a ProFlow Cell Filter (Bio-Rad, Hercules, CA) and subsequently stained with fluorescence-conjugated antibodies (FITC-conjugated anti-CD38, PE-conjugated anti-CD138; Beckman Coulter, Brea, CA). Plasma cells were isolated based on specific markers using an S3e™ Cell Sorter (Bio-Rad), with sorting efficacy confirmed by the presence of cells positive for CD38 and CD138, with a frequency exceeding 1%.

### Plasma cell sorting using MACS

EasySep™ Buffer and 1 x EasySep™ RBC lysis buffer were added to the cell pellet, followed by the introduction of antibody (anti-CD138) and beads (RapidSpheres™), with subsequent incubation. After adjusting the sample volume with EasySep™ buffer, it was placed onto “The Big Easy” EasySep™ magnet for incubation. The supernatant was removed and replaced with EasySep™ buffer. This process was repeated twice to ensure thorough purification. Finally, PBS was added, and the sample was vortexed, completing the bead sorting process for subsequent experiments.

### Plasma cell purity assessment

Microscopic examination and flow cytometry were used to compare sorting efficiency of two enrichment methods. For microscopic examination, smear slides from FACS- and MACS-sorted products of seven patients were stained with Wright-Giemsa, and plasma cells were counted microscopically by a pathologist. Plasma cell purity assessed by microscopic examination was defined as the percentage of plasma cells among all nucleated cells. For flow cytometry, the purity of FACS and MACS was compared using the S3e™ Cell Sorter (Bio-Rad). For FACS, cells that were positive for both CD38 and CD138 were isolated, collected in PBS, and re-analyzed with the S3e™ Cell Sorter (Bio-Rad) without further processing to assess purity. In the case of MACS, sorting products were stained using two fluorescence-conjugated antibodies (FITC-conjugated anti-CD38 and PE-conjugated anti-CD138; Beckman Coulter), following the same method used for FACS. Plasma cell purity assessed by flow cytometry was defined as percentage of CD38 + CD138 + cells among total cells.

### FISH using FACS-sorted cells

The sorted sample was initially dispensed onto a barrier slide and firmly affixed using an Epredia™ Cytospin™ 4 Cytocentrifuge (Thermo Fisher Scientific, Waltham, MA). Subsequently, the slide with the sorted sample was incubated in KCl (0.075 M) for 20 min at 38 °C. This step was followed by a 10-minute fixation period in Canoy’s Fixative solution. Once the slide had been thoroughly air-dried to eliminate residual moisture, probe attachment and the FISH test were carried out according to standard procedures.

### FISH using MACS-sorted cells

This FISH procedure involves cell harvest followed by fixation in Canoy’s Fixative solution. Subsequent centrifugation separates the fixed cells from the supernatant, which is then discarded. The turbidity of the resulting cell pellet is adjusted, and the sample is dispensed onto a barrier slide. Probe attachment ensues, facilitating the commencement of FISH testing in accordance with standardized protocols.

### Targeted panel sequencing

Genomic DNA was extracted from sorted products using a QIAamp DSP DNA Blood Mini Kit (Qiagen, Hilden, Germany). A custom capture panel (Dxome Co. Ltd., Gyeonggi-do, Korea) targeting coding exons and intron-exon boundaries of 112 genes related to lymphoid neoplasms (Supplementary Table S1) was used. Prepared libraries were hybridized with capture probes and sequenced as paired-end reads (2 × 150 bp) using NextSeq 550Dx (Illumina, San Diego, CA). NGS data analysis was performed with a DxSeq Analyzer (Dxome). Single-nucleotide variants (SNVs), small insertions and deletions (indels), and copy number variants (CNVs) were identified [8, 9]. All variants were classified using a four-tier system of the American College of Medical Genetics and Genomics, American Society of Clinical Oncology, and the College of American Pathologists [10].

### Statistical analysis

Statistical analysis was carried out using Analyse-it for Microsoft Excel 5.68 (Analyse-it Software Ltd, Leeds, UK) and Prism 8.0 (GraphPad Software, Inc, La Jolla, CA, USA). A Wilcoxon matched-pairs signed rank test was conducted to compare the plasma cell purity, percentages of abnormal cells from FISH, and variant allele frequencies (VAFs) from NGS between two enrichment methods. A *p* < 0.05 was considered statistically significant.

## Results

### Patient demographics

The demographic characteristics of 31 patients included in this study are presented in Supplementary Table S2 and are summarized in Table 1. The patients had a median age of 71 years with a slight male predominance (64.5%). Among them, 61.3% were diagnosed with MM, while 22.6% were diagnosed with MGUS.

**Table 1:** Demographic characteristics of 31 patients with plasma cell neoplasm

Variables	Median (IQR) or *N* (%)
Age, years	71 (58–75)
Male sex	20 (64.5)
Diagnosis	
Plasma cell myeloma	19 (61.3)
Monoclonal gammopathy of undetermined significance	7 (22.6)
Plasma cell myeloma s/p chemotherapy	2 (6.5)
Amyloidosis	1 (3.2)
Plasmacytoma	1 (3.2)
Plasma cell leukemia	1 (3.2)
Bone marrow plasma cell percentage	16.0 (7.6–39.4)
≥ 10%	21 (67.7)
< 10%	10 (32.3)
Immunological and biochemical markers	
Hb (g/dL)	10.8 (8.6–12.1)
Ca^2+^ (mg/dL)	9.0 (8.6–9.5)
Creatinine (mg/dL)	1.0 (0.9–1.8)
Albumin (g/dL)	3.8 (3.4–4.2)
β2-microglobulin (mg/dL)	3.6 (2.5–6.9)
Serum FLC ratio	2.0 (0.2–13.5)
Serum M-protein (g/dL)	0.9 (0.3–2.6)
Immunoglobulin type	
Heavy chain	
IgG	20 (64.5)
IgA	4 (12.9)
IgG and IgA	1 (3.2)
IgM	2 (6.5)
Light chain only	1 (3.2)
No monoclonal band	2 (6.5)
Light chain	
Kappa	16 (51.6)
Lambda	11 (35.5)
Lambda light chain disease IgG kappa and free lambda light chain	1 (3.2) 1 (3.2)
No monoclonal band	2 (6.5)
Karyotype	
Normal	21 (67.7)
Hyperdiploidy	7 (22.6)
Not interpretable	3 (9.7)

IQR, interquartile range

### Comparison of plasma cell purity between FACS and MACS

**Fig. 1:**
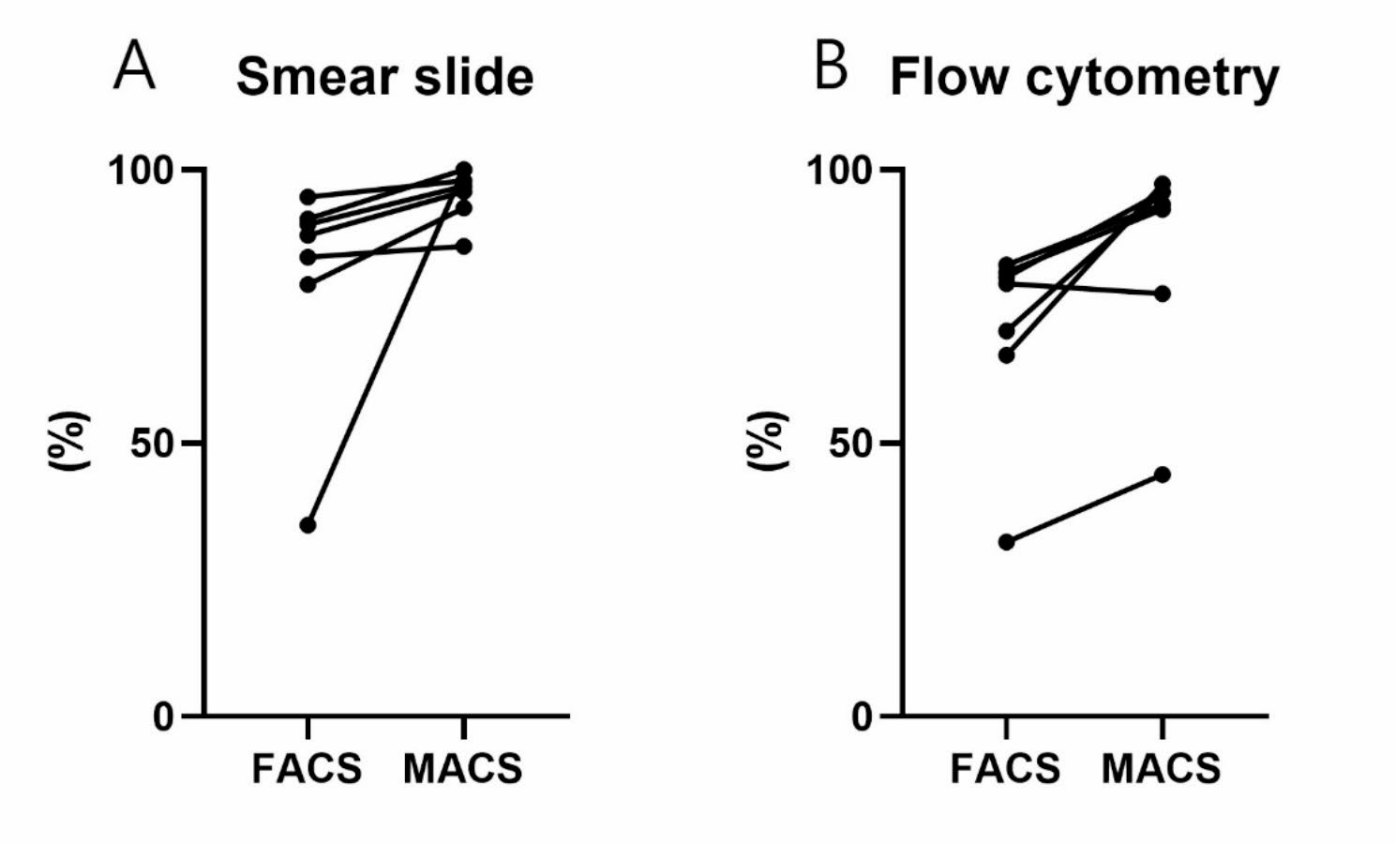
Comparison between plasma cell percentage of MACS-sorted samples versus FACS from (**A**) microscopic examination (Wilcoxon matched-pairs signed rank test, *p* = 0.0156) and (**B**) flow cytometry assessment (Wilcoxon matched-pairs signed rank test, *p* = 0.0313)

MACS-sorted samples yielded a higher percentage of plasma cells than FACS from microscopic examination (median of differences 7%, *p* = 0.0156) and flow cytometry assessment (median of differences 12.35%, *p* = 0.0313) (Fig. 1A and B). Selected slide images and flow cytometry results are presented in Supplementary Fig. S1.

### FISH

**Fig. 2:**
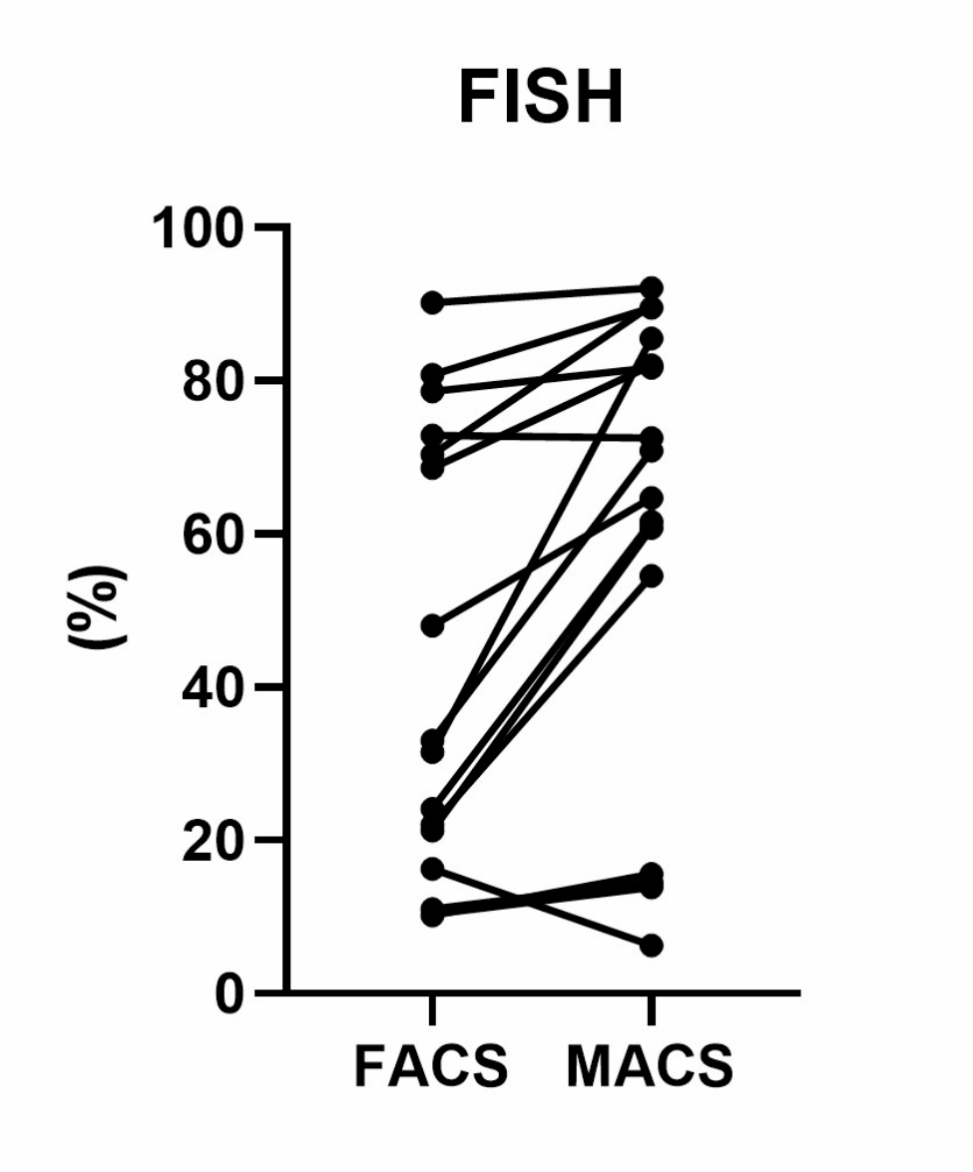
Percentage of abnormal cells from FISH according to enrichment methods. MACS-sorted samples showed significantly higher percentages of abnormal cells than FACS-sorted samples (Wilcoxon matched-pairs signed rank test, *p* = 0.0010)

Ten patient samples underwent both FACS and MACS for FISH analysis with five commercial probes, XL *TP53*/17cen, XL *FGFR3*/*IGH* DF, XL t(11;14) *CCND1*/*IGH* DF, XL t(14;16) *IGH*/*MAF* DF, and XL *CDKN2C*/*CKS1B* (all MetaSystems, Altlussheim, Germany). All tested samples showed good agreement between FACS and MACS (Supplementary Table S3). Sixteen abnormalities were found in tested samples, and the proportion of abnormal cells detected by FISH was significantly higher in MACS compared to FACS (median of differences 11.05%, *p* = 0.001, Fig. 2). This finding is consistent with the higher plasma cell purity with MACS than in FACS.

### NGS

**Fig. 3:**
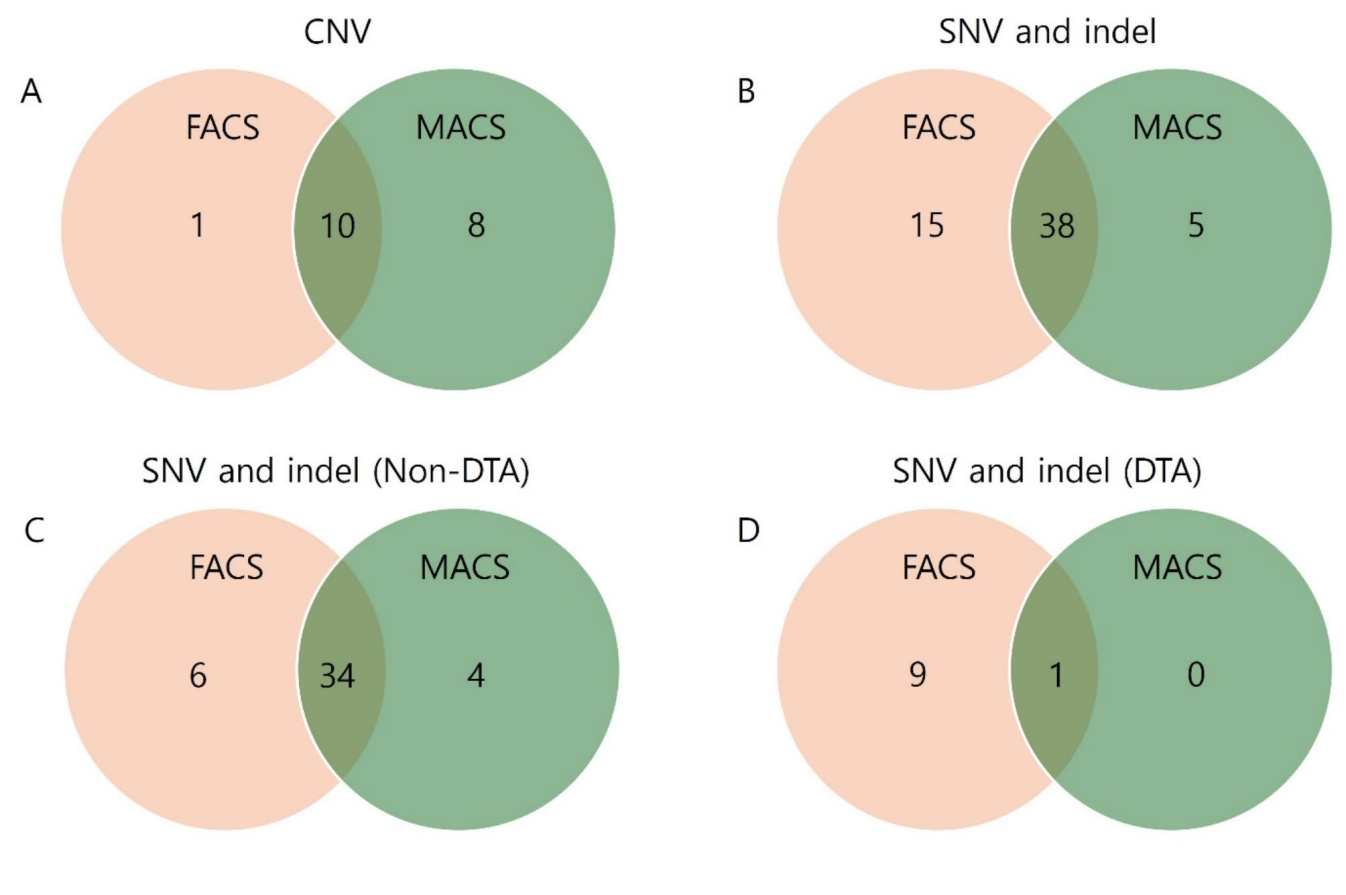
Concordance of NGS variants between FACS- and MACS-sorted samples (**A-D**). Patient 22 was excluded from DTA versus non-DTA gene analysis shown in panels **C** and **D**

**Fig. 4:**
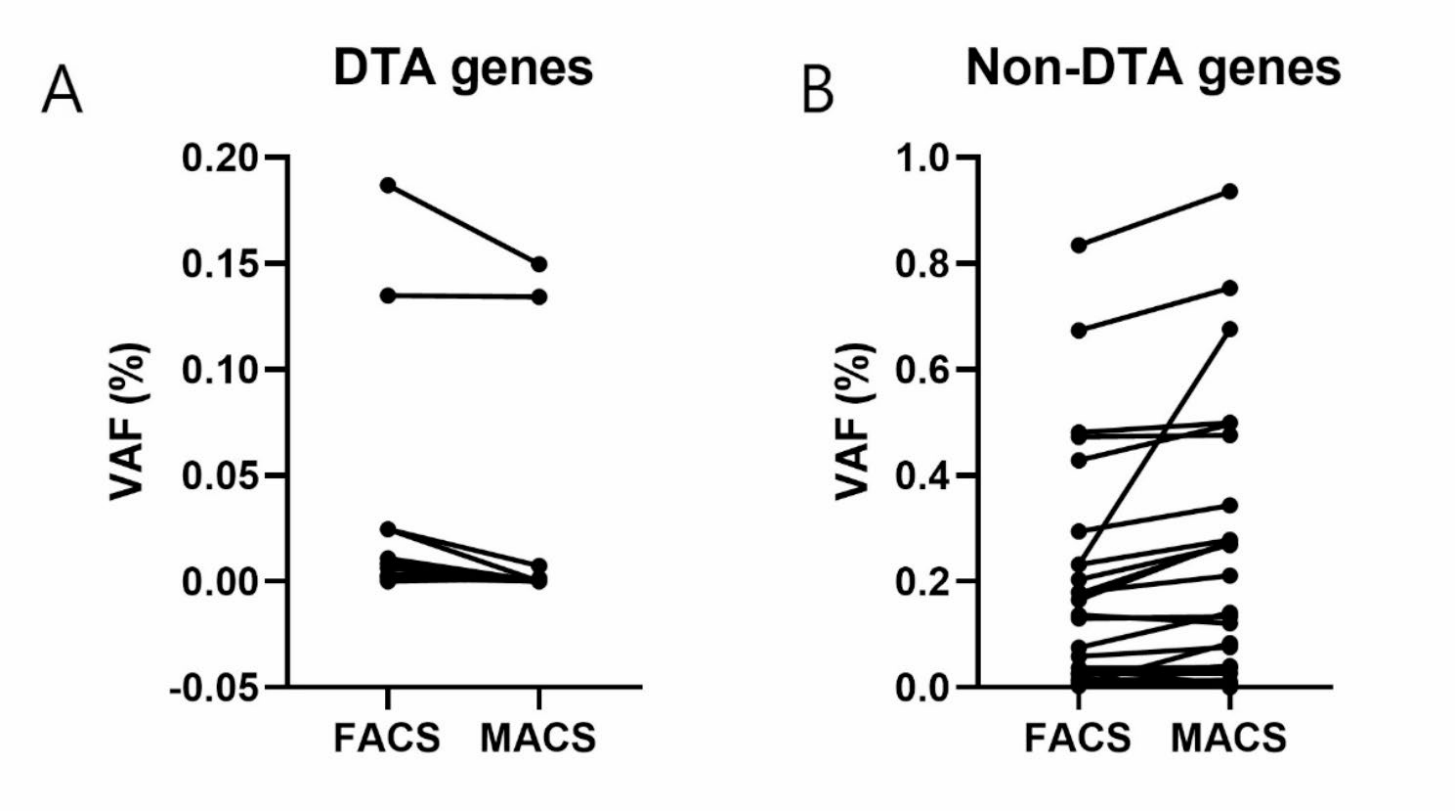
(**A**) Plot of the VAF of DTA genes using FACS- and MACS-sorted samples. (**B**) Plot of the VAF of non-DTA genes using FACS- and MACS-sorted samples. Patient 22 was excluded from this figure

Twenty-four of 31 patient samples were available for NGS analysis using FACS- and MACS-sorted products. Seventy-seven tier 1/2 variants were detected in at least one of the two tests. Forty-nine SNVs, nine indels, and 19 CNVs were identified in the following genes: *ARID1A*,* ASXL1*,* ATM*,* BIRC3*,* BRAF*,* BTG1*,* CDKN1B*,* CREBBP*,* DIS3*,* DNMT3A*,* IDH2*,* KRAS*,* MAP2K1*,* MGA*,* NRAS*,* PRDM1*,* PTEN*,* RB1*,* SF3B1*,* SGK1*,* TET2*,* TNFAIP3*,* TNFRSF14*,* TP53*, and *TRAF3*. All detected mutations and their corresponding VAFs from both FACS and MACS are detailed in Supplementary Table S4. Regarding CNVs, 10 were found in both FACS- and MACS-sorted samples, eight were identified only in MACS-sorted samples, and one was detected only in FACS-sorted samples (Fig. 3A). Regarding SNVs and indels, 38 variants were commonly identified in FACS- and MACS-sorted samples. Additionally, five variants were exclusively observed in MACS-sorted samples, while 15 were unique to FACS-sorted samples (Fig. 3B). Since the purity of the sorted product is less than 100%, we analyzed the presence and VAF trends of SNVs and indels by separating the *DNMT3A*, *TET2*, and *ASXL1* (DTA) genes (most commonly associated with clonal hematopoiesis) from other genes, assuming that clonal hematopoiesis variants may be detected alongside PCN-associated mutations. Mutations detected in patient 22 were excluded from the analysis, as the data did not reveal a dominant clone that could be identified as the myeloma clone. Thirty-four SNVs and indels in non-DTA genes were detected in both FACS- and MACS-sorted samples, while only one variant was commonly identified in the DTA genes across both methods (Fig. 3C and D). Wilcoxon matched-pairs signed rank test analysis for comparing VAFs from SNVs and indels showed that the median of differences (VAF of MACS minus VAF of FACS) in the DTA group was − 0.006555 (*p* = 0.0020), while that in the non-DTA group was 0.002805 (*p* = 0.0019) (Fig. 4).

## Discussion

Overall, our study demonstrated that MACS is as effective, if not slightly superior, to FACS in sorting plasma cells from bone marrow samples. Plasma cell purity from both microscopic examination and flow cytometry assessment consistently showed a higher percentage of plasma cells in MACS-sorted samples than FACS-sorted samples, aligning with similar findings using adipose tissue [7].

For FISH, the two sorting methods both effectively identified abnormal cytogenetic findings even in an MM patient (case 23) with low plasma cell (< 10%) percentage in aspiration. However, the percentage of abnormal cells varied between the methods, with MACS often showing higher abnormal cell percentages than FACS. This can lead to differences in detecting subclonal abnormalities, such as 17p deletion, or increased molecular heterogeneity [11].

The VAF of mutations detected by NGS was generally higher in the MACS-sorted samples than FACS-sorted samples. However, an interesting trend emerged between DTA and non-DTA groups. The VAFs of DTA genes, which account for up to 80% of clonal hematopoiesis of indeterminate potential (CHIP) mutations [12], were typically higher in FACS-sorted samples. This is consistent with the greater purity of plasma cells observed in our study. Additionally, more numerous CNVs were detected in MACS-sorted samples compared to FACS-sorted samples, highlighting the enhanced sensitivity of MACS in detecting genomic alterations.

The limitations of our study include a small sample size, with only a subset of samples undergoing plasma cell purity assessment, FISH, or NGS analysis. As a single-center study, the sample size was inherently limited. Additionally, tumor purity assessment bioinformatic tools, such as ASCAT, were not implemented, which may have impacted our ability to evaluate purity accurately. Finally, we did not confirm that the DTA gene mutations identified were definitively CHIP mutations, which could impact the interpretation of these findings. Comprehensive analysis involving large clinical samples could provide more solid evidence on optimal enrichment methods for PCN patients.

In conclusion, while FACS remains the gold standard for cell sorting, our study demonstrates that MACS is a viable alternative for plasma cell sorting in bone marrow samples of patients with PCN. Given the longer processing times and the need for specialized equipment associated with FACS, clinical laboratories can adopt MACS as an alternative method, particularly in settings where FACS equipment is unavailable or where faster processing is required, without compromising sorting effectiveness. Despite it can be useful to know the percentage of the population of interest before MACS.

## Electronic supplementary material

Below is the link to the electronic supplementary material.

Supplementary Material 1

## Data Availability

No datasets were generated or analysed during the current study.

## Abbreviations

CHIP: Clonal hematopoiesis of indeterminate potential
CNVs: Copy number variants
DTA: DNMT3A, TET2, and ASXL1
FISH: Fluorescence in situ hybridization
FACS: Fluorescence-activated cell sorting
MACS: Magnetic-activated cell sorting
MGUS: Monoclonal gammopathy of undetermined significance
MM: Multiple myeloma
PBS: Phosphate-buffered saline
PCN: Plasma cell neoplasm
SNVs: Single-nucleotide variants
Indels: Small insertions and deletions
VAF: Variant allele frequency
