# Supplementary material for: Evaluation of plasma cell sorting methods in multiple myeloma patients: flow cytometry versus magnetic beads
Source: Cancer Cell Int. 2025 Jan 17;25:16. doi: 10.1186/s12935-025-03647-8 (PMC11740577; doi:10.1186/s12935-025-03647-8)
Supplement: Supplementary file 1 — Supplementary Material 1 [file 12935_2025_3647_MOESM1_ESM.docx]

**Supplementary Figure S1.** Plasma cell yield assessment in a representative case. (A) Smear slides using FACS (right)- and MACS (left)-sorted samples Wright-Giemsa stained (case 8), looked under a microscope (x40). (B) Gating results of FACS (right)- and MACS (left)-sorted samples (case 5).


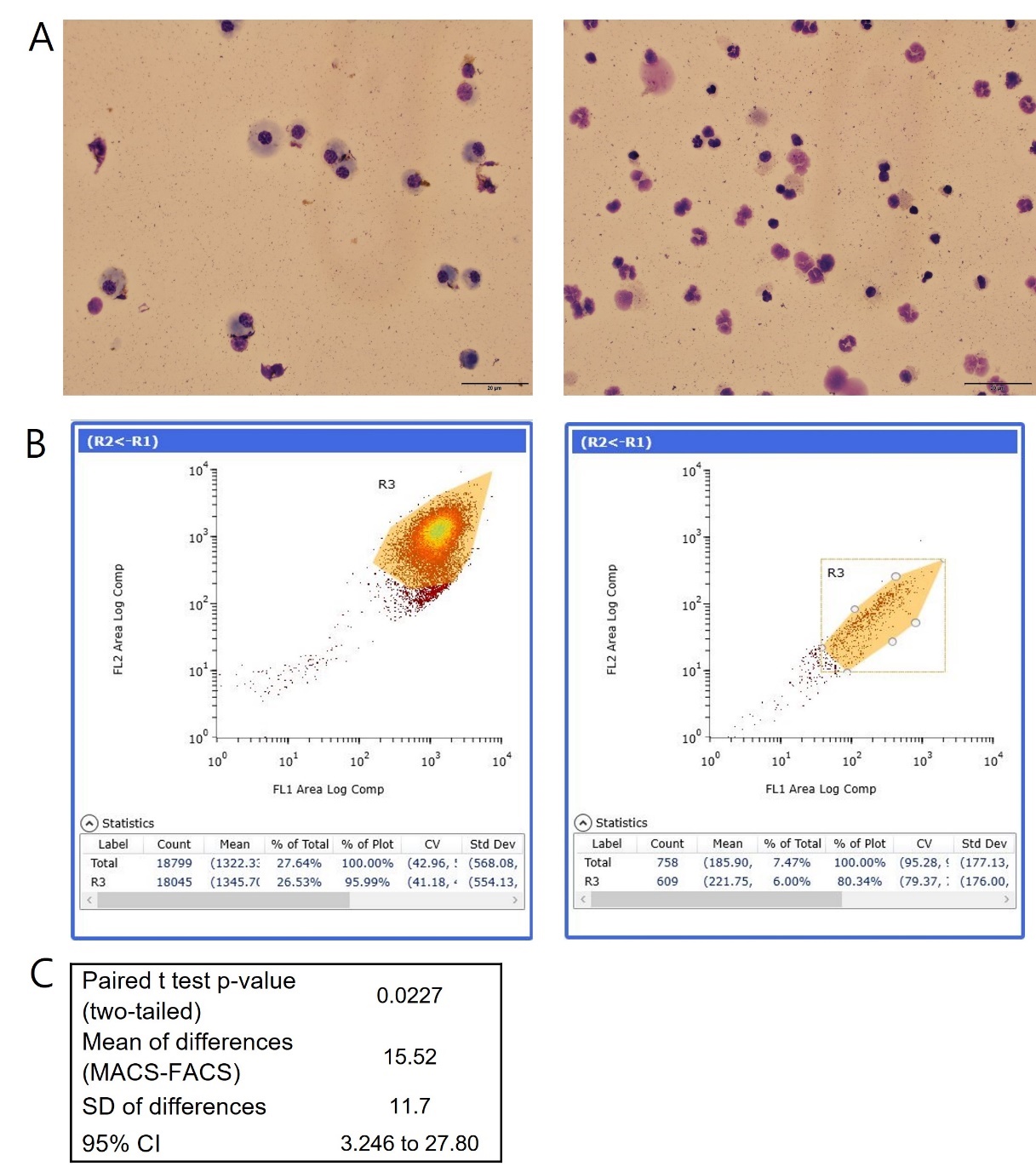


**Supplementary Table S1.** Genes included in the next-generation sequencing panel used in this study

| *ALK, ARID1A, ARID5B, ASXL1, ASXL3, ATM, B2M, BCL11B, BCL2, BCL6, BCOR, BCORL1, BIRC3, BIRC6, BRAF, BTG1, BTG2, BTK, CARD11, CCND1, CCND3, CD28, CD58, CD70, CD79A, CD79B, CDKN1B, CDKN2A, CDKN2B, CIITA, CREBBP, CXCR4, DDX3X, DIS3, DNMT3A, DTX1, DUSP22, EBF1, EGFR, EGR1, EP300, ETV6, EZH2, FAS, FBXW7, FOXO1, FYN, GNA13, H1-4, ID3, IDH2, IKZF3, IRF4, IRF8, ITPKB, JAK1, JAK3, KDM6A, KIT, KLHL14, KLHL6, KMT2A, KMT2C, KMT2D, KRAS, LRP1B, MAP2K1, MEF2B, MGA, MTOR, MYC, MYD88, NCOR1, NF1, NFKB2, NFKBIA, NOTCH1, NOTCH2, NRAS, NSD2, PAX5, PHF6, PIM1, PLCG1, PRDM1, PTEN, RB1, REL, RHOA, SETD1B, SETD2, SF3B1, SGK1, SMARCA4, SOCS1, SPEN, STAT3, STAT5B, STAT6, TBL1XR1, TCF3, TENT5C, TET1, TET2, TET3, TNFAIP3, TNFRSF14, TP53, TP63, TRAF3, UBR5, XPO1* |
| --- |

Supplementary Table S2. Demographic characteristics of 31 plasma cell neoplasm patients

| Patient ID | Sex | Age | Diagnosis | Bone marrow plasma cell (%) | Hb (g/dL) | Ca2+ (mg/dL) | Creatinine (mg/dL) | Albumin (g/dL) | β2-microglobulin (mg/dL) | Serum FLC ratio | Serum M-protein (g/dL) | Heavy-chain type | Light-chain type | Karyotype |
| --- | --- | --- | --- | --- | --- | --- | --- | --- | --- | --- | --- | --- | --- | --- |
| 01 | F | 73 | Plasma cell leukemia | 86 | 8.1 | 12.8 | 4.3 | 3.8 | 26.5 | 429.61 | 2.13 | IgG | kappa | 45,X,-X,der(8)t(8;16)(p11.2;p11.2),t(11;14)(q13;q32),-13,-16,+18,-22,+mar1,+mar2[18]/46,idem,+7[3] |
| 02 | M | 62 | Plasma cell myeloma | 91.9 | 10.8 | 9.7 | 1.21 | 3.9 | 6.08 | 0.01 | 4.35 | IgG | lambda | 51~56,XY,der(1)t(1;?)(p36.1;?),der(1)t(1;?)(p13;?),+5,-6,+9,+11,der(11)t(11;?)(p15;?),der(11)t(11;?)(q13;?),+15,+19,+21,+21,+mar[cp5]/46,XY[17] |
| 03 | M | 67 | Plasma cell myeloma | 34.2 | 12.9 | 8.9 | 0.94 | 4 | 2.23 | 0.98 | 1.66 | IgA | lambda | 56,XY,+dic(1;6)(p11;q14),+3,+5,+7,+9,+9,+15,+18,+19,+21[4]/46,XY[16] |
| 04 | M | 76 | Plasma cell myeloma | 70.4 | 10.9 | 9.2 | 1.01 | 3.7 | 4.76 | 313.3 | 3.82 | IgA | kappa | 46,XY[15] |
| 05 | F | 75 | Plasma cell myeloma | 86.1 | 5.8 | 8.5 | 1.97 | 3.2 | 12.4 | 202.99 | 5.06 | IgG | kappa | 50~53,X,-X,+5,der(6;12)(p10;q10),+der(7)t(7;?)(p11.2;?),del(7)(q32),+9,+11,+15,+19,+19,-20,+21,+mar[cp16]/46,XX[4] |
| 06 | M | 62 | Plasma cell myeloma | 16 | 12.1 | 8.6 | 0.88 | 3.9 | 2.46 | 0.02 | 0.51 | IgG | lambda | 46,XY[20] |
| 07 | F | 78 | Plasma cell myeloma | 98.4 | 7.2 | 9.7 | 1.36 | 3.2 | 11 | 0.01 | 3.57 | IgA | lambda | 53~55,X,-X,+der(1;16)(q10;?p10),der(2)t(2;?),der(4)t(4;?)(p14;?),+5,+der(6)t(6;?)(q21;?),+9,+11,+der(11)t(11;?)(p11.2;?),der(12)t(12;?)(p11.2;?),-13,+15,+15,+18,+1~2mar[cp19]/46,XX[1] |
| 08 | M | 61 | Plasma cell myeloma | 11.2 | 14.5 | 9 | 0.95 | 4.3 | 1.82 | 3.48 | 0.89 | IgG | kappa | 46,XY[20] |
| 09 | M | 75 | Monoclonal gammopathy of undetermined significance | 8.6 | 8.5 | 8 | 0.83 | 2.9 | - | 1.55 | 0.43 | IgG | kappa | 46,XY[20] |
| 10 | M | 54 | Plasma cell myeloma | 12.8 | 8.6 | 13.6 | 5.56 | 3.3 | 25.4 | 0.2 | 0.42 | IgA, IgG | lambda(beta region.) lambda(gamma region) | 46,XY[20] |
| 11 | M | 74 | Plasmacytoma | 3.2 | 14 | 9.2 | 0.76 | 4.5 | 1.94 | 10.36 | 0 | No monoclonal band | No monoclonal band | 46,XY[20] |
| 12 | M | 59 | Plasma cell myeloma | 25 | 13 | 9 | 0.88 | 4.1 | 2.09 | 0.03 | 1 | IgM | lambda | 46,XY[20] |
| 13 | M | 73 | Amyloidosis | 3.7 | 11.7 | 8.7 | 2.05 | 3.9 | 6.05 | 13.49 | 0.33 | IgM | kappa | Not interpretable result |
| 14 | M | 44 | Monoclonal gammopathy of undetermined significance | 0.3 | 13.2 | 8.6 | 0.66 | 4.1 | 1.25 | 2.37 | 0 | IgG | kappa | 46,XY[20] |
| 15 | F | 84 | Lymphoma-associated monoclonal gammopathy | 2.5 | 9.6 | 8.4 | 1.06 | 3.6 | - | 1.97 | 1.04 | IgG | kappa | 46,XX[20] |
| 16 | F | 57 | Plasma cell myeloma | 24 | 10.8 | 9.5 | 0.68 | 5 | 1.99 | 0.06 | 0 | - | Lambda | 46,XX[11] |
| 17 | M | 81 | Plasma cell myeloma | 40.9 | 5.8 | 9 | 1.59 | 2.7 | 22.5 | <0.01 | 0.46 | IgA | lambda(beta region.) lambda(gamma region) | Not interpretable results |
| 18 | F | 73 | Known plasma cell myeloma s/p chemotherapy | 18 | 11.9 | 9.2 | 1.07 | 4 | 2.91 | 22.23 | 1.25 | IgG | kappa | 46,XX[20] |
| 19 | M | 39 | Plasma cell myeloma | 14.6 | 15.7 | 9.6 | 0.91 | 5 | 1.46 | <0.01 | 0.08 | IgD | lambda | 46,XY[20] |
| 20 | F | 71 | Plasma cell myeloma | 31.9 | 11.7 | 8.7 | 1.01 | 3.7 | 3.85 | 0.17 | 2.49 | IgG | lambda | 46,XX[20] |
| 21 | M | 73 | Plasma cell myeloma | 87.4 | 8.3 | 8.5 | 1.97 | 2.9 | 7.9 | 0.01 | 2.87 | IgG | lambda | 53,XY,+3,+5,+7,+9,der(9;12)(q10;q10),?der(14),+der(15)t(1;15)(q11;p11-13),+19,+19,+21[12]/55,XY,+3,+5,+6,+7,+9,+der(15)t(1;15),+19,+19,+21[4]/46,XY[4] |
| 22 | M | 83 | Plasma cell neoplasm | 10.5 | 8.4 | 8.4 | 2.42 | 3.7 | 3.56 | 11.52 | 0.54 | IgG | kappa | 46,XY[20] |
| 23 | M | 78 | Plasma cell myeloma | 9 | 10.6 | 8.6 | 0.63 | 4.4 | 2.53 | 0.01 | 0.04 | IgG | kappa Lambda paraprotein (light chain disease) | 46,XY[20] |
| 24 | F | 47 | Monoclonal gammopathy of undetermined significance | 3 | 7.7 | 9.6 | 5.68 | 3.2 | 2.59 | 1.29 | 0.32 | IgG | kappa | 46,XX[20] |
| 25 | F | 52 | Monoclonal gammopathy of undetermined significance | 7 | 13.9 | 9.2 | 0.74 | 4.3 | 3.91 | 2.15 | 0 | No monoclonal band | No monoclonal band | 46,XX[20] |
| 26 | M | 67 | Plasma cell myeloma | 21.3 | 12 | 8.6 | 0.73 | 3.8 | 3.04 | 85.75 | 4.29 | IgG | kappa | 46,XY[20] |
| 27 | F | 41 | Monoclonal gammopathy of undetermined significance | 8.1 | 9.1 | 9.2 | 0.45 | 3.5 | 2.83 | 0.83 | 0.47 | IgG | lambda | 46,XX[20] |
| 28 | M | 75 | Plasma cell myeloma | 37.8 | 11.7 | 10.1 | 2.12 | 4.3 | 4.15 | 198.9 | 2.8 | IgG | kappa | 45,X,-Y[17]/46~52,XY,-1,-4,+5,-7,+9,-10,+11,+15,+18,+19,-20,+1~4mar[cp3] |
| 29 | M | 74 | Plasma cell myeloma | 53.8 | 9.1 | 8.3 | 1.19 | 3.5 | 6.89 | 375.24 | 3.85 | IgG | kappa | 46,XY[20] |
| 30 | F | 35 | Monoclonal gammopathy of undetermined significance | 5.8 | 8.8 | 6.2 | 1.07 | 2.6 | 11.3 | 0.7 | 0.09 | IgG | kappa | 46,XX[20] |
| 31 | M | 64 | Plasma cell myeloma s/p autologous hematopoietic stem cell transplantation | 1 | 11.2 | 9.5 | 0.95 | 4.3 | 2.58 | 5.49 | 1.39 | IgG | kappa | Not interpretable results |

Supplementary Table S3. FISH results from 10 plasma cell neoplasm patients

| **Patient ID** | **BM plasma cell (%)** | **Method** | **FISH results*** | | | | |
| --- | --- | --- | --- | --- | --- | --- | --- |
|  |  |  | ***TP53*/17cen** | ***FGFR3*/*IGH*** | ***CCND1*/*IGH*** | ***IGH*/*MAF*** | ***CDKN2C*/*CKS1B*** |
| 20 | 31.9 | FACS | Negative | nuc ish(*FGFR3*x2,*IGH*x3)[175/240] | nuc ish(*CCND1*,*IGH*)x3(*CCND1* con *IGH*x2)[187/238] | nuc ish(*IGH*x3,*MAF*x1)[219/311] | Negative |
|  |  | MACS | Negative | nuc ish(*FGFR3*x2,*IGH*x3)[137/189] | nuc ish(*CCND1*,*IGH*)x3(*CCND1* con *IGH*x2)[125/153] | nuc ish(*IGH*x3,*MAF*x1)[231/257] | Negative |
| 21 | 87.4 | FACS | Negative | Negative | Negative | Negative | nuc ish(*CDKN2C*x2,*CKS1B*x3)[267/296] |
|  |  | MACS | Negative | Negative | Negative | Negative | nuc ish(*CDKN2C*x2,*CKS1B*x3)[209/227] |
| 23 | 9 | FACS | Negative | nuc ish(*FGFR3*x2,*IGH*x3)[35/319] | nuc ish(*CCND1*x2,*IGH*x3)[22/216] | nuc ish(*IGH*x3,*MAF*x2)[26/250] | nuc ish(*CDKN2C*x2,*CKS1B*x3~4)[98/311] |
|  |  | MACS | Negative | nuc ish(*FGFR3*x2,*IGH*x3)[48/331] | nuc ish(*CCND1*x2,*IGH*x3)[52/334] | nuc ish(*IGH*x3,*MAF*x2)[46/332] | nuc ish(*CDKN2C*x2,*CKS1B*x3~4)[318/372] |
| 24 | 3 | FACS | Negative | Negative | Negative | Negative | Negative |
|  |  | MACS | Negative | Negative | Negative | Negative | Negative |
| 25 | 7 | FACS | Negative | Negative | Negative | Negative | Negative |
|  |  | MACS | Negative | Negative | Negative | Negative | Negative |
| 26 | 21.3 | FACS | Negative | nuc ish(*FGFR3*,*IGH*)x3(*FGFR3* con *IGH*x2)[48/199] | nuc ish(*CCND1*x2,*IGH*x3)[54/245] | nuc ish(*IGH*x3,*MAF*x2)[52/244] | nuc ish(*CDKN2C*x2,*CKS1B*x3)[75/227] |
|  |  | MACS | Negative | nuc ish(*FGFR3*,*IGH*)x3(*FGFR3* con *IGH*x2)[122/198] | nuc ish(*CCND1*x2,*IGH*x3)[122/224] | nuc ish(*IGH*x3,*MAF*x2)[175/288] | nuc ish(*CDKN2C*x2~3,*CKS1B*x3)[124/175] |
| 27 | 8.1 | FACS | Negative | Negative | Negative | Negative | Negative |
|  |  | MACS | Negative | Negative | Negative | Negative | Negative |
| 28 | 37.8 | FACS | nuc ish(TP53x1,CEP17x2)[33/203] | nuc ish(*FGFR3*x2,*IGH*x3)[78/147] | nuc ish(*CCND1*x3,*IGH*x3)[94/137] | nuc ish(*MAF*x2,*IGH*x3)[72/150] | Negative |
|  |  | MACS | nuc ish(TP53x1,CEP17x2)[16/254] | nuc ish(*FGFR3*x2,*IGH*x3)[123/183] | nuc ish(*CCND1*x3,*IGH*x3)[114/139] | nuc ish(*IGH*X3,*MAF*x2)[101/156] | Negative |
| 29 | 53.8 | FACS | Negative | Negative | nuc ish(*CCND1*X3,*IGH*x2)[105/130] | Negative | Negative |
|  |  | MACS | Negative | Negative | nuc ish(*CCND1*X3,*IGH*x2)[111/124] | Negative | Negative |
| 31 | 1 | FACS | Negative | Negative | Negative | Negative | Negative |
|  |  | MACS | Negative | Negative | Negative | Negative | Negative |

FISH panel was done with five commercial probes, including XL *TP53*/17cen, XL *FGFR3*/*IGH* DF, XL t(11;14) *CCND1*/*IGH* DF, XL t(14;16) *IGH*/*MAF* DF, and XL *CDKN2C*/*CKS1B* (all MetaSystems, Altlussheim, Germany).

ND, not done

Supplementary Table S4. All detected mutations and their corresponding variant allele frequencies from both FACS and MACS

| Patient ID | Gene | HGVSc | HGVSp | VAF (FACS) | VAF (MACS) |
| --- | --- | --- | --- | --- | --- |
| 1 | *TP53* | c.658T>A | p.Tyr220Asn | 0.48092 | 0.49931 |
| 1 | *TP53* | c.524G>A | p.Arg175His | 0.47287 | 0.47658 |
| 1 | *NRAS* | c.181C>A | p.Gln61Lys | 0.02685 | 0.02708 |
| 1 | *NRAS* | c.183A>C | p.Gln61His | 0.03736 | 0.03674 |
| 1 | *NRAS* | c.182A>G | p.Gln61Arg | 0.01615 | 0.0113 |
| 1 | *NRAS* | c.37G>C | p.Gly13Arg | 0.00325 | ND |
| 1 | *NRAS* | c.38G>A | p.Gly13Asp | ND | 0.00269 |
| 1 | *KRAS* | c.53C>A | p.Ala18Asp | 0.13034 | 0.13346 |
| 1 | *KRAS* | c.183A>C | p.Gln61His | 0.03736 | 0.005 |
| 1 | *RB1* | Whole gene deletion |  | D | D |
| 1 | *DIS3* | Whole gene deletion |  | D | D |
| 2 | *NRAS* | c.35G>A | p.Gly12Asp | 0.8349 | 0.93634 |
| 3 | *BRAF* | c.1790T>G | p.Leu597Arg | 0.1371 | 0.12115 |
| 3 | *BRAF* | c.1514T>A | p.Leu505His | 0.00681 | 0.00456 |
| 3 | *KRAS* | c.190T>G | p.Tyr64Asp | 0.0262 | 0.027 |
| 3 | *KRAS* | c.182A>G | p.Gln61Arg | 0.00409 | 0.00442 |
| 3 | *NRAS* | c.34G>C | p.Gly12Arg | 0.008 | 0.01327 |
| 3 | *NRAS* | c.38G>A | p.Gly13Asp | 0.025 | 0.04083 |
| 4 | *KRAS* | c.437C>T | p.Ala146Val | 0.17953 | 0.21092 |
| 4 | *KRAS* | c.182A>G | p.Gln61Arg | 0.05854 | 0.07611 |
| 4 | *KRAS* | c.38G>A | p.Gly13Asp | 0.00728 | 0.00517 |
| 4 | *SF3B1* | c.1997A>T | p.Lys666Met | 0.23178 | 0.27888 |
| 5 | *TET2* | c.1648C>T | p.Arg550Ter | 0.01104 | ND |
| 5 | *SGK1* | Whole gene deletion |  | D | D |
| 5 | *PRDM1* | Whole gene deletion |  | D | D |
| 5 | *TNFAIP3* | Whole gene deletion |  | D | D |
| 6 | *CREBBP* | c.3917_3920del | p.Phe1306CysfsTer6 | ND | 0.00292 |
| 6 | *PRDM1* | Whole gene deletion |  | ND | D |
| 6 | *TNFAIP3* | Whole gene deletion |  | ND | D |
| 6 | *SGK1* | Whole gene deletion |  | ND | D |
| 7 | *RB1* | c.297G>A | p.Trp99Ter | 0.00863 | 0.00857 |
| 7 | *RB1* | c.1393G>T | p.Glu465Ter | 0.16537 | 0.2759 |
| 7 | *KRAS* | c.38G>A | p.Gly13Asp | 0.29483 | 0.34362 |
| 7 | *KRAS* | c.35G>T | p.Gly12Val | 0.00537 | 0.0838 |
| 7 | *BTG1* | c.133C>T | p.Gln45Ter | 0.42843 | 0.4961 |
| 7 | *ASXL1* | c.3162_3163insCCAA | p.Arg1055ProfsTer33 | 0.003 | ND |
| 7 | *TET2* | c.1723del | p.Ala575ArgfsTer5 | 0.00632 | ND |
| 7 | *DNMT3A* | c.1668G>C | p.Arg556Ser | 0.02475 | ND |
| 7 | *RB1* | Whole gene deletion |  | D | D |
| 7 | *DIS3* | Whole gene deletion |  | D | D |
| 7 | *BRAF* | Whole gene duplication/amplification | | D | D |
| 7 | *IRF8* | Whole gene deletion |  | D | D |
| 8 | *ATM* | c.7889T>A | p.Leu2630Ter | 0.00216 | ND |
| 8 | *MAP2K1* | c.361T>A | p.Cys121Ser | 0.03542 | 0.03567 |
| 9 | *BIRC3* | c.1299_1302del | p.Arg434GlnfsTer12 | ND | 0.00192 |
| 10 | *-* |  |  |  |  |
| 11 | *-* |  |  |  |  |
| 12 | *BRAF* | c.1789C>G | p.Leu597Val | 0.00184 | ND |
| 12 | *TNFRSF14* | Whole gene deletion |  | D | ND |
| 12 | *CDKN1B* | Whole gene deletion |  | D | D |
| 12 | *RB1* | Whole gene deletion |  | ND | D |
| 13 | *-* |  |  |  |  |
| 14 | *-* |  |  |  |  |
| 15 | *IDH2* | c.419G>A | p.Arg140Gln | 0.00868 | ND |
| 16 | *KRAS* | c.64C>A | p.Gln22Lys | 0.00759 | ND |
| 16 | *RB1* | Whole gene deletion |  | ND | D |
| 17 | *-* |  |  |  |  |
| 18 | *TP53* | c.841G>A | p.Asp281Asn | 0.23244 | 0.6766 |
| 18 | *TP53* | c.499C>T | p.Gln167Ter | 0.01233 | 0.01305 |
| 18 | *PTEN* | c.655C>T | p.Gln219Ter | 0.00153 | ND |
| 18 | *DNMT3A* | c.2622T>A | p.Tyr874Ter | 0.02471 | 0.00731 |
| 18 | *ASXL1* | c.2427del | p.Asp810ThrfsTer8 | 0.00358 | ND |
| 18 | *TET2* | c.817C>T | p.Gln273Ter | 0.0085 | ND |
| 18 | *TET2* | c.3315_3316insAA | p.Glu1106LysfsTer8 | 0.00184 | ND |
| 19 | *ARID1A* | c.2077C>T | p.Arg693Ter | 0.02461 | 0.04001 |
| 19 | *ARID1A* | c.6143G>A | p.Trp2048Ter | 0.07555 | 0.1409 |
| 19 | *DNMT3A* | c.994G>A | p.Gly332Arg | 0.00233 | ND |
| 20 | *KRAS* | c.35G>T | p.Gly12Val | 0.01031 | 0.00918 |
| 20 | *TRAF3* | c.487C>T | p.Arg163Ter | 0.20365 | 0.26853 |
| 20 | *TRAF3* | c.854del | p.Asn285ThrfsTer38 | 0.17707 | 0.27235 |
| 20 | *TRAF3* | c.1063dup | p.Glu355GlyfsTer35 | 0.01073 | 0.01384 |
| 20 | *BRAF* | c.1780G>A | p.Asp594Asn | 0.00544 | 0.00526 |
| 20 | *NRAS* | c.34G>C | p.Gly12Arg | ND | 0.00857 |
| 21 | *KRAS* | c.38G>A | p.Gly13Asp | 0.67361 | 0.75385 |
| 22 | *ASXL1* | c.1762C>T | p.Gln588Ter | 0.18695 | 0.1497 |
| 22 | *MGA* | c.6061_6062del | p.Leu2021GlufsTer2 | 0.03659 | 0.03808 |
| 22 | *TET2* | c.4122T>A | p.Cys1374Ter | 0.13498 | 0.13424 |
| 22 | *TET2* | c.5158A>T | p.Lys1720Ter | ND | 0.00152 |
| 23 | *DNMT3A* | c.1554+1G>A |  | 0.00679 | ND |
| 23 | *RB1* | Whole gene deletion |  | ND | D |
| 23 | *DIS3* | Whole gene deletion |  | ND | D |
| 23 | *CDKN1B* | Whole gene deletion |  | ND | D |
| 24 | - |  |  |  |  |

VAF, variant allele frequency; D, detected; ND, not detected
